# Clinicopathological and prognostic value of transforming acidic coiled-coil-containing protein 3 (TACC3) expression in soft tissue sarcomas

**DOI:** 10.1371/journal.pone.0188096

**Published:** 2017-11-14

**Authors:** Kotaro Matsuda, Hiroaki Miyoshi, Koji Hiraoka, Shintaro Yokoyama, Toshiaki Haraguchi, Toshihiro Hashiguchi, Tetsuya Hamada, Naoto Shiba, Koichi Ohshima

**Affiliations:** 1 Department of Pathology, Kurume University School of Medicine, Kurume, Fukuoka, Japan; 2 Department of Orthopedic Surgery, Kurume University School of Medicine, Kurume, Fukuoka, Japan; 3 Department of Surgery, Kurume University School of Medicine, Kurume, Fukuoka, Japan; University of South Alabama Mitchell Cancer Institute, UNITED STATES

## Abstract

Transforming acidic coiled-coil-containing protein 3 (TACC3), a microtubule regulator, is associated with various cancers. However, the relationship between TACC3 and soft tissue sarcomas (STS) remains unclear. We investigated the expression of TACC3 in 136 STS patient samples using immunohistochemical (IHC) staining, and the statistical associations between TACC3 expression and clinicopathological characteristics were evaluated. Additionally, the expression levels of the tumor suppressor p53 and of the cell proliferation marker Ki-67 were also assessed by IHC. High TACC3 expression was detected in 94/136 of STS cases (69.1%), and significantly correlated with higher grade according to the French Fédération Nationale des Centres de Lutte Contre le Cancer system (P<0.0001), poorer tumor differentiation (P<0.0001), increased mitotic counts (P<0.0001), advanced stage per American Joint Committee on Cancer guidelines (P<0.0001), higher p53 expression (P = 0.0487), higher Ki-67 expression (P<0.0001), and undergoing postoperative therapy (P = 0.0001). Disease-free survival (DFS) and overall survival (OS) of patients with high TACC3 expression were significantly shorter (P<0.0001 and P<0.0001, respectively). On multivariate analyses, high TACC3 expression was an independent negative prognostic factor for both DFS and OS (hazard ratio [HR]: 3.074; P = 0.0235 and HR: 8.521; P = 0.0415, respectively). Our results suggest that TACC3 is an independent prognostic factor and may be a novel therapeutic target for the treatment of STS.

## Introduction

Soft tissue sarcomas (STS) are rare, and comprise a heterogeneous group of mesenchymal tumors. Although they account for less than 1% of all human malignancies [1], they exhibit very aggressive behavior; approximately half of the patients with STS experience tumor recurrence despite undergoing multimodal therapy, and more than one-third of patients die of their diseases [2]. Complete resection is the first-line treatment, and there are few confirmed treatment options available other than conventional chemotherapy [3, 4]. Thus, the diagnosis and treatment of STS remain challenging for both physicians and patients.

Transforming acidic coiled-coil-containing protein 3 (TACC3) is a member of the TACC family, which is located in the 4p16.3 region of chromosome [5]. TACC3, an Aurora A kinase target, is essential for microtubule growth and stability when localizing to the centrosome during mitosis [6]. Microtubule fusion is necessary for the formation of the mitotic spindles, that segregate chromosomes. Abnormality of microtubules/centrosomes causes mitotic spindle defects and correlates with tumorigenesis and tumor progression; therefore, the involvement of TACC3 in various cancers has been reported [7]. Additionally, TACC3 depletion has been shown to induce p53-mediated apoptosis [8]. Despite the recent development of chemotherapeutic drugs that target mitotic proteins such as Aurora kinase and Polo-like kinase, their effects remain ambiguous [9]. Accordingly, TACC3 itself is being examined as a potentially novel therapeutic target.

To date, high TACC3 expression has been shown to be associated with poor prognosis in several cancers such as non-small cell lung cancer [10, 11], esophageal squamous cell carcinoma [12], hepatocellular carcinoma [13, 14], gastric cancer [15], colorectal cancer [16], breast cancer [17], and cholangiocarcinoma [18]. However, the clinicopathological and prognostic importance of TACC3 expression in STS has not been investigated.

In this study, we investigated TACC3 expression in STS using immunohistochemical (IHC) staining, and analyzed the association between TACC3 expression and clinicopathological characteristics as well as patient survival.

## Material and methods

### Patients and characteristics

We investigated 136 STS patients who underwent resection at Kurume University between 1990 and 2016, and retrieved formalin-fixed, paraffin-embedded (FFPE) specimens. The antibodies used for diagnoses were CAM5.2 (BD Biosciences, San Jose, CA, USA); CD3, MyoD1, Bcl-6, and CD34 (Leica Ltd., New Castle, UK); Bcl-2 and p16 (Ventana Medical Systems, Inc., Tucson, AZ, USA); MDM2 (Calbiochem, San Diego, CA, USA); CDK4 (Invitrogen, San Francisco, CA, USA); and vimentin, SMA, desmin, myogenin, S100, KP-1, AE1/AE3, EMA, CD20, and CD99 (Dako, Tokyo, Japan). Samples were reviewed and classified by 2 experienced pathologists (O.K. and M.H.) according to the 2013 World Health Organization (WHO) classification [1]. Patients with microscopic positive surgical margins, those with recurrence or metastasis at the initial visit, or those who received neoadjuvant therapies were excluded from the study. All patients underwent postoperative follow-up at least every other year after surgery. Clinical data were collected from patients’ medical records. Our study was approved by the Research Ethics Committee of Kurume University, and written informed consent was obtained according to the Declaration of Helsinki.

All STS samples were cut into maximum cross sections and stained with hematoxylin and eosin for pathomorphological investigation. We selected sites that were most representative of the sample’s pathological features, as determined by the 2 pathologists, and constructed tissue microarrays from 3-mm cores with a minimum of 2 cores per patient. If tissues were available, 3 cores per patient were used to compensate for STS tissue heterogeneity.

Based on previous studies [19–23], patients were classified by sex, age (≤60 or >60 years), tumor size (≤5 cm or >5 cm), depth of tumor (superficial or deep), tumor location (extremities or trunk), the French Fédération Nationale des Centres de Lutte Contre le Cancer system (FNCLCC) grade (grade 1 or 2 or 3), tumor differentiation (score 1 or 2 or 3), mitotic count (0–9/10 or ≥10/10 high-power fields [HPF]), tumor necrosis (<50% or ≥50%), the American Joint Committee on Cancer (AJCC) stage (I or II or III), p53 expression (low or high), Ki-67 expression (low or high), and treatment (surgery alone or combined with postoperative therapy).

### Evaluation of TACC3, p53, and Ki-67

#### Immunohistochemical staining

After deparaffinization, antigen retrieval, and endogenous peroxidase blocking, FFPE tissues of 3-μm thicknesses were incubated with TACC3 (rabbit monoclonal, clone: EPR7756; ab134154 [Abcam], 1:200 dilution), p53 (mouse monoclonal, clone: Do-7; M7001 [Dako]), and Ki-67 (mouse monoclonal, clone: MIB-1; M7240, [Dako]) as primary antibodies. Samples were next incubated with Dako REAL EnVision Detection System (Dako) as a secondary antibody. Diaminobenzidine was used to visualize the immunoreaction.

The cytoplasmic staining was defined as TACC3-positive according to the criteria used in other malignancies [10–18]. Furthermore, nuclear staining was regarded as positive for p53 and Ki-67 based on previous studies [19, 21, 22]. The ratios of TACC3-, p53-, and Ki-67-positive tumor cells were calculated by manual counting of at least 100 tumor cells in 5 fields under an optical microscope with 400-fold magnification by the 2 pathologists, who were blinded to any clinical details.

#### Determination of cut-off values

**Fig 1:**
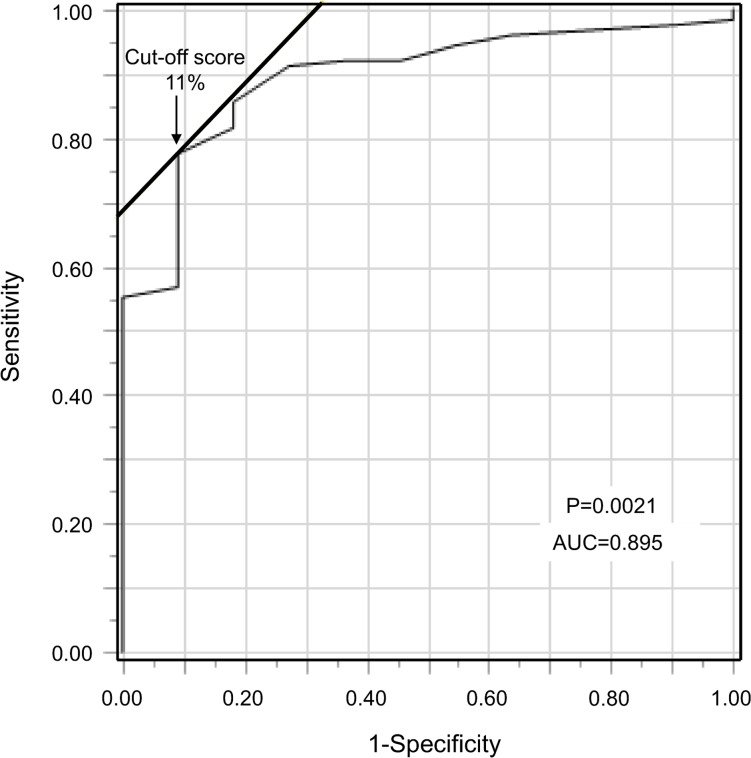
Receiver operating characteristic curve. The cut-off value for determining TACC3 positivity was 11% according to the Youden index. AUC: area under the curve.

The cut-off value for TACC3 expression was determined by using receiver operating characteristic (ROC) curves and calculating the Youden index (Fig 1). FNCLCC grades were used as dichotomous variables (either 1 or 2/3), while the TACC3-positive rate was a continuous variable. The optimal cut-off value was calculated as 11%. Therefore, TACC3-positive rates of more than 11% were defined as high expression. Based on previous studies, high expression rates of p53 and Ki-67 were defined as greater than 10% positive staining [19, 21, 22].

### Statistical analysis

Clinicopathological features and TACC3 expression were analyzed using the χ^2^ or Fisher’s exact test. Disease-free survival (DFS) was defined as the duration between the day resection was performed and the day recurrence or metastasis was detected; overall survival (OS) was defined as the duration between the day resection was performed and the day of last follow-up or death. Both DFS and OS were evaluated using the Kaplan-Meier method and log-rank test. The impact of possible risk factors affecting DFS and OS were assessed by univariate and multivariate Cox regression analyses. A P-value less than 0.05 was considered statistically significant. The JMP, version 12 software (SAS Institute, Tokyo, Japan) was used for statistical analyses in this study.

## Results

### Clinicopathological characteristics of patients

Patients’ characteristics and histological types according to the WHO histological classification are shown in Tables 1 and 2, respectively. Total of 73 males (53.7%) and 63 females (46.3%) were included. They were ranged from 8–95 years (mean 58.4 years). The mean value of tumor size was 7.86 cm (range 1–25 cm). There were 53 superficial tumors (39.0%), whereas 83 deep tumors (61.0%). In total, 107 tumors (78.7%) originated in the extremities, whereas 29 (21.3%) in the trunk. According to FNCLCC grade, patients with grade 1 were 11 cases (8.1%), grade 2 were 49 cases (36.0%), and grade 3 were 76 cases (55.9%). According to AJCC stage, patients with stage IA were 6 cases (4.4%), stage IB were 5 cases (3.7%), stage IIA were 35 cases (25.7%), stage IIB were 35 cases (25.7%), and stage III were 55 cases (40.4%). High expression of p53 and Ki-67 were observed in 67 cases (49.3%) and 69 cases (50.7%), respectively. In addition to resection, 22 patients (16.2%) received chemotherapy, 22 patients (16.2%) received radiotherapy, and 25 patients (18.4%) received chemoradiotherapy. Sixty-three patients (46.3%) experienced local recurrence or distant metastasis, and 39 patients (28.7%) died of their tumors during the follow-up period, which ranged from 2–279 months (mean 50.7 months).

**Table 1:** Clinicopathological characteristics of patients.

Characteristics	ALL (n = 136)	(%)
Sex		
Male	73	53.7%
Female	63	46.3%
Age, mean (range), years	58.4 (8–95)	
≤60	66	48.5%
>60	70	51.5%
Tumor size, mean (range), cm	7.86 (1–25)	
≤5	46	33.8%
>5	90	66.2%
Depth		
Superficial	53	39.0%
Deep	83	61.0%
Tumor location		
Extremities	107	78.7%
Trunk	29	21.3%
FNCLCC		
1	11	8.1%
2	49	36.0%
3	76	55.9%
Tumor differentiation		
1	2	1.5%
2	53	39.0%
3	81	59.5%
Mitotic count		
0–9/10 HPF	29	21.3%
10–19/10 HPF	72	52.9%
>19/10 HPF	35	25.7%
Tumor necrosis		
No necrosis	23	16.9%
<50%	109	80.1%
≥50%	4	2.9%
AJCC		
IA	6	4.4%
IB	5	3.7%
IIA	35	25.7%
IIB	35	25.7%
III	55	40.4%
p53 expression		
Low	67	49.3%
High	69	50.7%
Ki-67 expression		
Low	60	44.1%
High	76	55.9%
Therapy		
Surgery	67	49.3%
Surgery followed by chemotherapy	22	16.2%
Surgery followed by radiotherapy	22	16.2%
Surgery followed by chemoradiotherapy	25	18.4%
Follow up, mean (range), month	50.7 (2–279)	
Recurrence or metastasis		
Absent	73	53.7%
Present	63	46.3%
Termination		
Alive	97	71.3%
Dead	39	28.7%

FNCLCC = French Fédération Nationale des Centres de Lutte Contre le Cancer; AJCC = American Joint Committee on Cancer; TACC3 = Transforming acidic coiled-coil-containing protein 3

HPF = high-power fields.

**Table 2:** Histological types of STS.

Histological type	All (n = 136) (%)
Undifferentiated/unclassified sarcomas	37 (27.2%)
Myxoid liposarcoma	16 (11.7%)
Pleomorphic liposarcoma	4 (2.9%)
Dedifferentiated liposarcoma	3 (2.2%)
Leiomyosarcoma	19 (14.0%)
Myxofibrosarcoma	15 (11.0%)
Malignant peripheral nerve sheath tumor	10 (7.4%)
Fibrosarcoma	8 (5.9%)
Synovial sarcoma	7 (5.1%)
Extraskeletal osteosarcoma	6 (4.4%)
Alveolar soft part sarcoma	5 (3.7%)
Rhabdomyosarcoma	3 (2.2%)
Epithelioid sarcoma	2 (1.5%)
Angiosarcoma	1 (0.7%)

STS = Soft tissue sarcomas.

### Assessment of TACC3 expression

**Fig 2:**
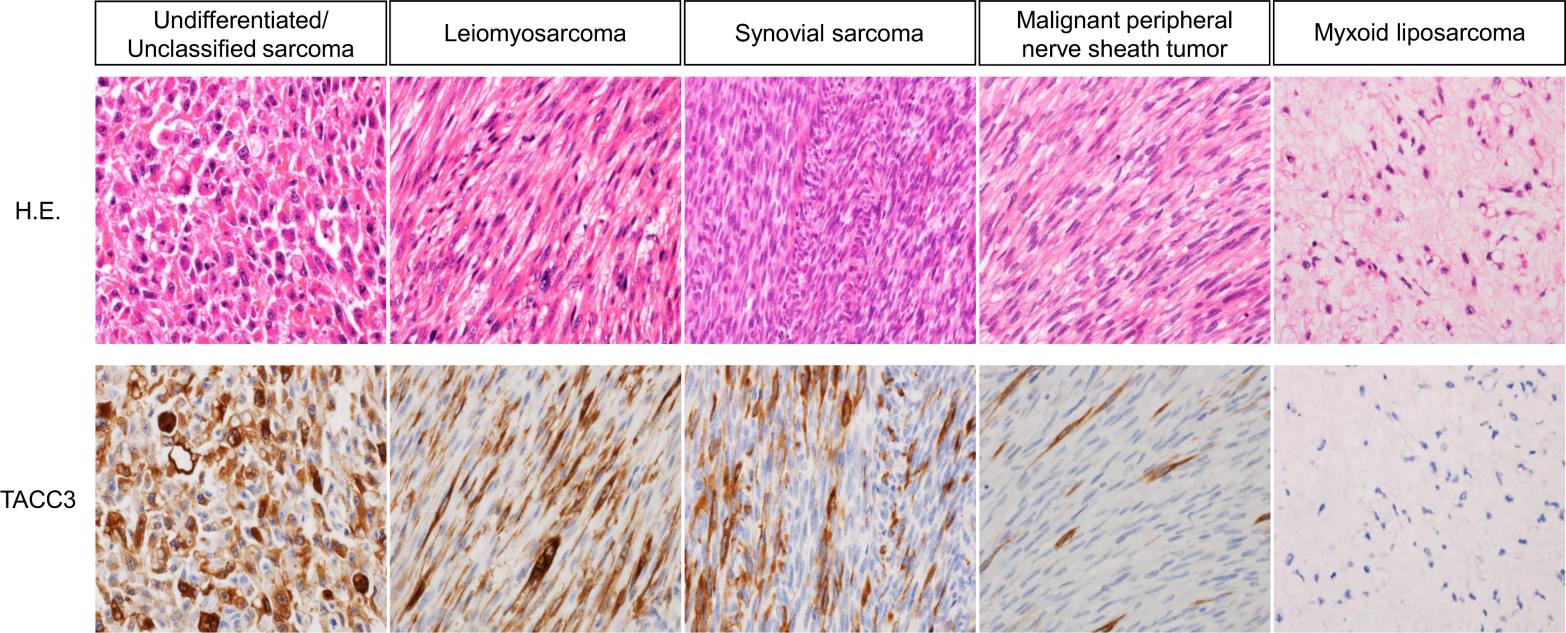
Immunohistochemical staining for TACC3 in representative images of soft tissue sarcomas. Columns 1–5 show, respectively, an undifferentiated/unclassified sarcoma case with 80% TACC3 expression, a leiomyosarcoma case with 60% TACC3 expression, a synovial sarcoma case with 40% TACC3 expression, a malignant peripheral nerve sheath tumor case with 10% TACC3 expression, and a myxoid liposarcoma case with 0% TACC3 expression. (H.E. = hematoxylin and eosin; original magnification, ×400).

Typical expression patterns of STS are shown in Fig 2. While TACC3 was highly expressed in 94/136 cases (69.1%), the expression of TACC3 was recognized in the cytoplasm of tumor cells in all cases. There was no particular pattern of association between TACC3 expression and morphological and immunohistochemical findings.

With respect to histological types, high TACC3 expression was observed in 30/37 of undifferentiated/unclassified sarcomas (81.1%), 4/16 of myxoid liposarcomas (25.0%), 4/4 of pleomorphic liposarcomas (100%), 2/3 of dedifferentiated liposarcomas (66.7%), 18/19 of leiomyosarcomas (94.7%), 9/15 of myxofibrosarcomas (60.0%), 7/10 of malignant peripheral nerve sheath tumors (70.0%), 2/8 of fibrosarcomas (25.0%), 4/7 of synovial sarcomas (57.1%), 6/6 of extraskeletal osteosarcomas (100%), 2/5 of alveolar soft part sarcomas (40.0%), 3/3 of rhabdomyosarcomas (100%), 2/2 of epithelioid sarcomas (100%), and 1/1 angiosarcoma (100%).

### Association of TACC3 expression with clinicopathological features

The statistical associations of TACC3 expression with specific clinicopathological features are shown in Table 3. Higher TACC3 expression was significantly associated with higher FNCLCC grade (P<0.0001), poorer tumor differentiation (P<0.0001), increased mitotic counts (P<0.0001), advanced AJCC stage (P<0.0001), higher p53 expression (P = 0.0487), higher Ki-67 expression (P<0.0001), and presence of postoperative therapy (P = 0.0001). On the other hand, there were no significant differences in terms of sex (P = 0.566), age (P = 0.548), tumor size (P = 0.273), tumor depth (P = 0.368), tumor location (P = 0.821), or tumor necrosis (P = 0.311).

**Table 3:** Statistical association of TACC3 expression with clinicopathological features.

Characteristics	TACC3 high expression n = 94 (69.1%)	TACC3 low expression n = 42 (30.9%)	P-value
Sex			0.566
Male	52 (55.3%)	21 (50.0%)	
Female	42 (44.7%)	21 (50.0%)	
Age (years)			0.548
≤60	44 (46.8%)	22 (52.4%)	
>60	50 (53.2%)	20 (47.6%)	
Tumor size (cm)			0.273
≤5	29 (30.8%)	17 (40.5%)	
>5	65 (69.2%)	25 (59.5%)	
Depth			0.368
Superficial	39 (41.5%)	14 (33.3%)	
Deep	55 (58.5%)	28 (66.7%)	
Tumor location			0.821^a^
Extremities	73 (77.7%)	34 (80.9%)	
Trunk	21 (22.3%)	8 (19.1%)	
FNCLCC			<0.0001*^a^
1	1 (1.0%)	10 (23.8%)	
2	26 (27.7%)	23 (54.8%)	
3	67 (71.3%)	9 (21.4%)	
Tumor differentiation			<0.0001*
1	0 (0%)	2 (4.8%)	
2	27 (28.7%)	26 (61.9%)	
3	67 (71.3%)	14 (33.3%)	
Mitotic count			<0.0001*^a^
0–9/10 HPF	8 (8.5%)	22 (52.4%)	
≥10/10 HPF	86 (91.5%)	20 (47.6%)	
Tumor necrosis			0.311^a^
<50%	90 (95.7%)	42 (100%)	
≥50%	4 (4.3%)	0 (0%)	
AJCC			<0.0001*^a^
I	1 (1.0%)	10 (23.8%)	
II	44 (46.8%)	26 (61.9%)	
III	49 (52.1%)	6 (14.3%)	
p53 expression			0.0487*
Low	41 (43.6%)	26 (61.9%)	
High	53 (56.4%)	16 (38.1%)	
Ki-67 expression			<0.0001*^a^
Low	23 (24.5%)	37 (88.1%)	
High	71 (75.5%)	5 (11.9%)	
Treatment			0.0001*
Surgery alone	36 (38.3%)	31 (73.8%)	
Postoperative therapy	58 (61.7%)	11 (26.2%)	

* P-value<0.05

^a^ P-value calculated by Fisher's exact test.

TACC3 = Transforming acidic coiled-coil-containing protein 3; FNCLCC = French Fédération Nationale des Centres de Lutte Contre le Cancer; AJCC = American Joint Committee on Cancer; HPF = high-power fields.

### Effect of TACC3 expression on DFS and OS

**Fig 3:**
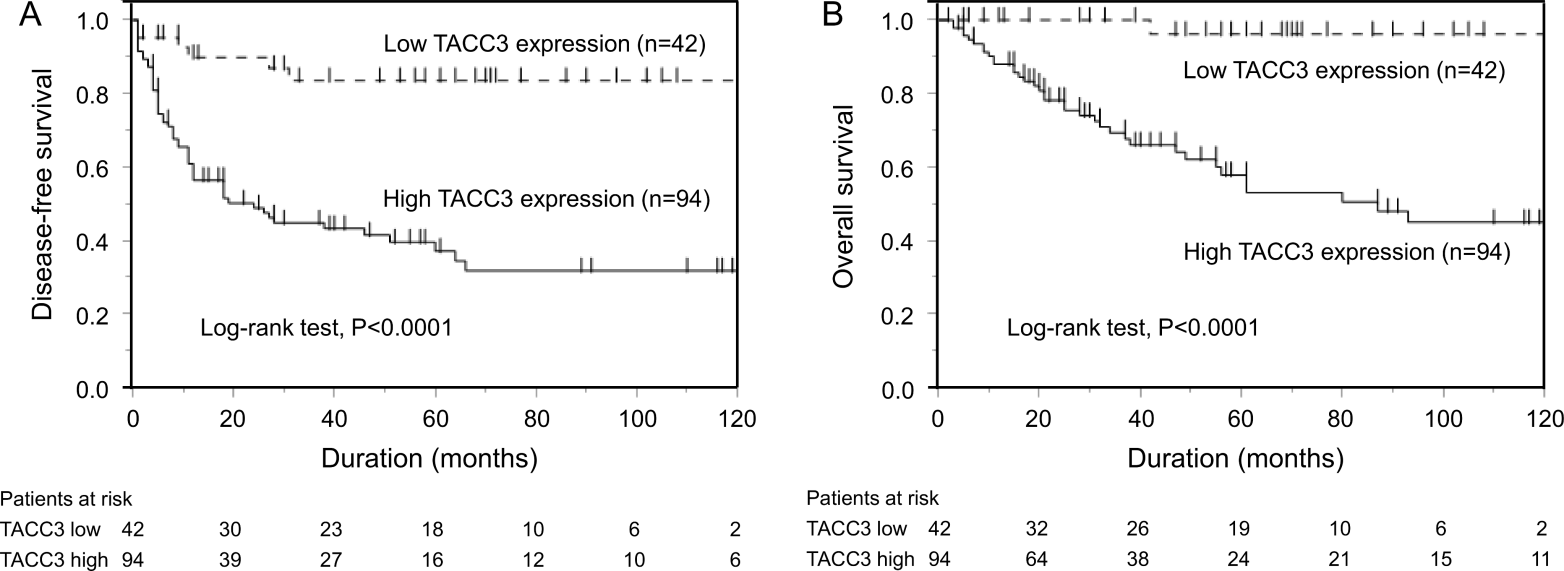
Kaplan-Meier curves for disease-free survival (DFS) and overall survival (OS) of patients with soft tissue sarcomas. (A) The DFS of patients with high TACC3 expression was significantly worse (P<0.0001). (B) The OS of patients with high TACC3 expression was also significantly worse (P<0.0001).

DFS and OS curves of patients with STS are shown in Fig 3. DFS in patients with high TACC3 expression was significantly shorter than in those with low TACC3 expression (P<0.0001) (Fig 3A), as was OS (P<0.0001) (Fig 3B).

### Risk factors affecting DFS and OS

The results of univariate and multivariate analyses of potential risk factors that influence DFS and OS are shown in Tables 4 and 5, respectively. Larger tumor size (hazard ratio [HR]: 1.053; P = 0.0290); a higher FNCLCC grade (HR: 2.061 [2 vs. 1], 4.442 [3 vs. 1], 2.156 [3 vs. 2]; P = 0.0063); greater expression levels of TACC3 (HR: 4.762; P = 0.0001), p53 (HR: 1.707; P = 0.0402), and Ki-67 (HR: 1.877; P = 0.0180); and undergoing postoperative therapy (HR: 3.783; P<0.0001) were negative risk factors for DFS on univariate analysis, while greater TACC3 expression level (HR: 3.074; P = 0.0235) and undergoing postoperative therapy (HR: 2.540; P = 0.0030) were independent negative prognostic factors for DFS on multivariate analysis. Furthermore, a higher FNCLCC grade (HR: 3.283 [3 vs. 2]; P = 0.0180); greater expression levels of TACC3 (HR: 20.206; P = 0.0030), p53 (HR: 2.508; P = 0.0101), and Ki-67 (HR: 1.949; P = 0.0484); and undergoing postoperative therapy (HR: 5.204; P = 0.0002) were negative risk factors for OS on univariate analysis, whereas greater TACC3 expression level (HR: 8.521; P = 0.0415) was an independent negative prognostic factor for OS on multivariate analysis.

**Table 4:** Univariate and multivariate Cox regression analyses of risk factors affecting DFS in STS patients.

	Univariate analysis		Multivariate analysis	
Characteristics	Hazard ratio (95% confidence interval)	P-value	Hazard ratio (95% confidence interval)	P-value
Sex, male (vs. female)	1.003 (0.610–1.668)	0.991		
Age (years)	1.003 (0.990–1.018)	0.644		
Tumor size (cm)	1.053 (1.003–1.100)	0.0290*	1.023 (0.972–1.072)	0.358
Depth, deep (vs. superficial)	0.809 (0.490–1.354)	0.412		
Tumor location, trunk (vs. extremities)	1.033 (0.548–1.823)	0.916		
FNCLCC, 2 (vs. 1)	2.061 (0.586–13.037)	0.0063*	0.860 (0.206–5.863)	0.341
3 (vs. 1)	4.442 (1.370–27.238)		1.340 (0.327–9.114)	
3 (vs. 2)	2.156 (1.243–3.935)		1.558 (0.878–2.905)	
TACC3 expression, high (vs. low)	4.762 (2.313–11.511)	0.0001*	3.074 (1.239–8.851)	0.0235*
p53 expression, high (vs. low)	1.707 (1.032–2.881)	0.0402*	1.120 (0.657–1.941)	0.680
Ki-67 expression, high (vs. low)	1.877 (1.126–3.214)	0.0180*	0.909 (0.520–1.651)	0.745
Treatment, postoperative therapy (vs. surgery alone)	3.783 (2.168–7.000)	<0.0001*	2.540 (1.404–4.847)	0.0030*

* P-value<0.05.

DFS = Disease free survival; STS = Soft tissue sarcomas; FNCLCC = French Fédération Nationale des Centres de Lutte Contre le Cancer; AJCC = American Joint Committee on Cancer; TACC3 = Transforming acidic coiled-coil-containing protein 3.

**Table 5:** Univariate and multivariate Cox regression analyses of risk factors affecting OS in STS patients.

	Univariate analysis		Multivariate analysis	
Characteristics	Hazard ratio (95% confidence interval)	P-value	Hazard ratio (95% confidence interval)	P-value
Sex, male (vs. female)	1.245 (0.658–2.434)	0.507		
Age (years)	1.004 (0.987–1.023)	0.633		
Tumor size (cm)	1.050 (0.987–1.108)	0.101		
Depth, deep (vs. superficial)	0.871 (0.462–1.681)	0.672		
Tumor location, trunk (vs. extremities)	1.399 (0.667–2.733)	0.346		
FNCLCC, 2 (vs. 1)	Unavailable data**	0.0180*	Unavailable data**	0.208
3 (vs. 1)	Unavailable data**		Unavailable data**	
3 (vs. 2)	3.283 (1.530–8.129)		2.126 (0.997–5.321)	
TACC3 expression, high (vs. low)	20.206 (4.377–358.778)	0.0030*	8.521 (1.645–156.898)	0.0415*
p53 expression, high (vs. low)	2.508 (1.279–5.277)	0.0101*	1.618 (0.809–3.455)	0.190
Ki-67 expression, high (vs. low)	1.949 (1.019–3.879)	0.0484*	0.926 (0.469–1.926)	0.829
Treatment, postoperative therapy (vs. surgery alone)	5.204 (2.340–13.813)	0.0002*	2.461 (0.997–6.709)	0.0507

* P-value<0.05

**Hazard ratio was too huge since no patient with FNCLCC grade 1 experienced tumor death.

OS = overall survival; STS = Soft tissue sarcomas; FNCLCC = French Fédération Nationale des Centres de Lutte Contre le Cancer; AJCC = American Joint Committee on Cancer; TACC3 = Transforming acidic coiled-coil-containing protein 3.

## Discussion

Our study revealed that TACC3 was highly expressed in more than two-thirds of STS patients, and that greater expression was associated with higher FNCLCC grade, poorer tumor differentiation, increased mitotic counts, advanced AJCC stage, higher p53 expression, higher Ki-67 expression, and undergoing postoperative therapy. The DFS and OS rates in STS patients with high TACC3 expression were significantly shorter. Furthermore, on multivariate analyses, high TACC3 expression was an independent negative prognostic factor for both DFS and OS in STS patients.

Higher TACC3 expression was associated with poorer tumor differentiation of FNCLCC in STS. The relationship between higher TACC3 expression and poorer tumor differentiation was demonstrated in lung cancer [11], hepatocellular carcinoma [14], and cholangiocarcinoma [18]. In addition, TACC3 expression was suggested to be accelerated during the transition of breast cancer from ductal carcinoma in situ to invasive ductal carcinoma in microarray analysis [24]. Even though simple comparison is difficult due to the heterogeneity of STS, it is possible that, in STS, TACC3 is involved in the tumor differentiation as with other malignant tumors.

Higher TACC3 expression was associated with increased mitotic counts, and TACC3 was detected in almost all mitotic tumor cells by IHC (S1 Fig). These results are consistent with the known mechanism of TACC3 as a protein that is expressed specifically in the mitotic phase [5, 7].

However, TACC3 was also expressed in many non-mitotic tumor cells on IHC. In normal human tissues, TACC3 expression is observed in the testis, spleen, thymus, and peripheral blood leukocytes, all of which are highly proliferative tissues [25]. Additionally, TACC3 expression is reportedly correlated with proliferation in hepatocellular carcinoma cells [14]. In our study, greater expression of TACC3 was associated with that of Ki-67. Ki-67 is a nuclear antigen that is expressed throughout the cell cycle except during G0 phase, and is reflective of the cell’s proliferative potential [26]. Therefore, our results suggest that TACC3 expression is a hallmark of STS cell proliferation.

Higher TACC3 expression was associated with increased p53 expression; patients with high p53 expression tended to have poor prognoses, which is consistent with previous findings [19, 21]. Generally, missense *p53* gene mutations result in the accumulation of p53 proteins in the nuclei. On the other hand, *p53* mutation is not always correlated with IHC findings, because mutant protein staining differs by both site and extent [27]. However, it was previously reported that *p53* mutation correlates with nuclear immunoreaction of p53 protein in STS [21]; therefore, high TACC3 expression may indeed correlate with *p53* mutation in STS.

High TACC3 expression was an independent predictor of poor prognosis in terms of both DFS and OS; this may be attributed to at least 3 possible explanations. First, overexpression of TACC3 promotes the PI3K/AKT and ERK signaling pathways that induce epithelial-mesenchymal transition, which enhances the migratory ability of tumor cells and increases their invasive capacity [28]. Second, TACC3 expression was associated with the expression of stem cell transcription factors, including Bmi-1, c-Myc, and Nanog, and the upregulation of TACC3 promoted tumor stem cell-like capabilities in hepatocellular carcinoma [13]; cancer stem cells can maintain themselves by self-replication to create additional daughter cells in their milieus [29]. Third, high levels of TACC3 reduce the efficiency of DNA repair, causing genomic instability and tumorigenesis [30]. However, p53-mediated apoptosis may not function since it is induced by the lack of TACC3 [8]. Taken together, high TACC3 expression may increase the aggressiveness of tumor cells by increasing their invasive capacities, stem cell-like properties, and antiapoptotic activities.

The relationships between high TACC3 expression and poor prognoses have been reported in various cancers to date [10–18]; however, cut-off values for high TACC3 on IHC have not been clarified. In non-small cell carcinoma, the cut-off value of TACC3 was defined as 10% [10]. In a study of esophageal squamous cell carcinomas, however, the cut-off value for high TACC3 expression was determined by the immunoreactivity score, product of the staining intensity, and percentage of positive cells [12]; moreover, the rationale behind the selected cut-off value was not stated. In our case, we determined the cut-off value via ROC curve analysis and the Youden index using the FNCLCC grade, established grade of malignancy, and indicators of STS prognosis [20] as dichotomous variables, as well as the TACC3 positivity rate as a continuous variable. Hence, our derived cut-off value was not arbitrary, and should therefore be considered sound [31].

The depletion of TACC3 has been shown to suppress tumor growth without damaging normal tissues [32], cause premature senescence of tumor cells [33], and increase the susceptibility of tumor cells to paclitaxel (which disrupts the microtubule network) [34]. Furthermore, TACC3 inhibitors such as spindlactone (SPL) and SNIPER(TACC3) have been developed [35, 36]. SPL interferes with TACC3 function by blocking the TACC3-TOGp complex and causing spindle defects in tumor cells, whereas SNIPER(TACC3) reduces TACC3 protein levels in tumor cells by inducing its polyubiquitylation and proteasomal degradation. These developments improve the prospects of TACC3 as a novel therapeutic target.

Our study has some limitations. First, the number of patients and histological types were relatively small. There are numerous histological types of STS, and further studies with larger numbers of patients that encompass these histological types are required to clarify common progression mechanisms. At the same time, STS are rare, and many studies included even fewer patients than ours, even though our results were ultimately consistent with theirs [19, 21]. Second, TACC3 expression was evaluated only by IHC; additional investigations of genetic defects and transcriptional systems ought to reveal other biological roles for TACC3. Third, postoperative therapy was associated with high TACC3 expression and also correlated with shorter DFS and OS. These results suggest that surgeons might administer adjuvant treatment to more aggressive or higher-grade tumors. Our study was based on a retrospective analysis, which may be susceptible to selection bias. Therefore, prospective studies are required to validate our results.

In conclusion, we demonstrated that high TACC3 expression was correlated with aggressive clinicopathological features and unfavorable prognoses in STS patients. To our knowledge, ours is the first study of its kind to investigate TACC3 in STS. Our results expose the potential of TACC3 not only as a reliable prognostic biomarker but also as a novel and promising therapeutic target for the treatment of STS.

## Supporting information

S1 Fig TACC3 expression of mitotic tumor cells. Almost all mitotic tumor cells are stained with TACC3. (A) A case of undifferentiated/unclassified sarcoma. (B) A case of leiomyosarcoma. (C) A case of synovial sarcoma. (D) A case of malignant peripheral nerve sheath tumor. (original magnification, ×600).
(TIF)

S1 Table Data analyzed in this study. (XLSX)
