# Supplementary figures and images for: Clinicopathological and prognostic value of transforming acidic coiled-coil-containing protein 3 (TACC3) expression in soft tissue sarcomas
Source: PLoS One. 2017 Nov 14;12(11):e0188096. doi: 10.1371/journal.pone.0188096 (PMC5685599; doi:10.1371/journal.pone.0188096)

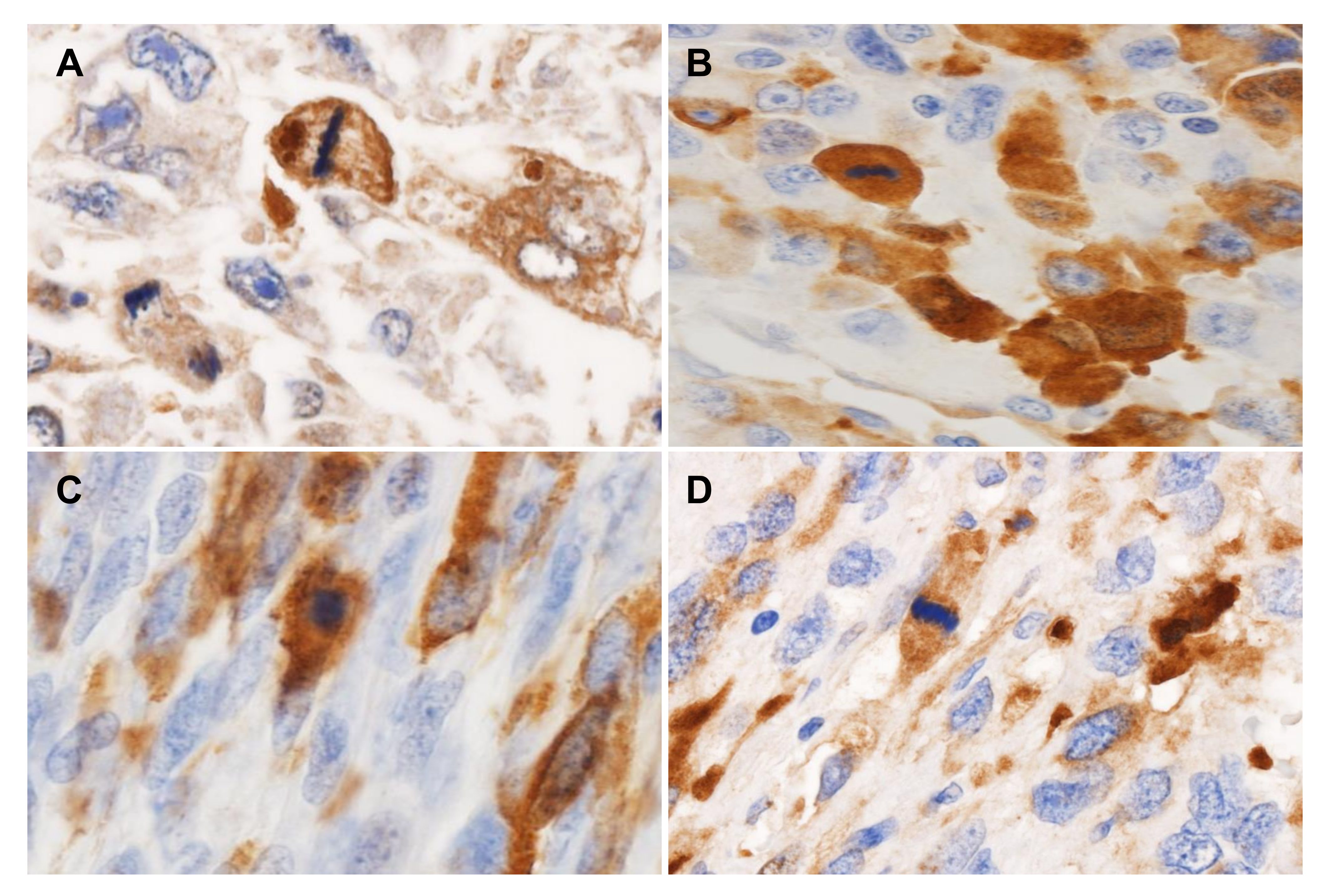

Supplement: S1 Fig — Almost all mitotic tumor cells are stained with TACC3. (A) A case of undifferentiated/unclassified sarcoma. (B) A case of leiomyosarcoma. (C) A case of synovial sarcoma. (D) A case of malignant peripheral nerve sheath tumor. (original magnification, ×600). (TIF) [file pone.0188096.s001.tif]
